# Hypertension among Mongolian adults in China: A cross‐sectional study of prevalence, awareness, treatment, control, and related factors: Hypertension among Mongolian adults in China

**DOI:** 10.1111/jch.14348

**Published:** 2021-08-16

**Authors:** Peiyao Yu, Yuzhen Ning, Yumin Gao, Yanping Zhao, Lin Tie, Lijitu Wu, Lili Zhang, Ru Zhang, Meng Cui, Hui Pang, Qian Wu, Zhidi Wang, Le Chen, Lingyan Zhao

**Affiliations:** ^1^ Inner Mongolia Medical University Hohhot China; ^2^ Laboratory for Molecular Epidemiology in Chronic Diseases Inner Mongolia Medical University Hohhot China; ^3^ Affiliated Hospital of Inner Mongolia Medical University Hohhot China; ^4^ Health Center of Bayanmangha Daolaodu Sumu Jarud Banner Tongliao China; ^5^ Community Health Service Center of Shangdu Town Zhenglan Banner Xilingol League China; ^6^ Xilinhot Community Health Service Center of Chugulan Xilingol League China; ^7^ Shanxi Medical University Taiyuan China

**Keywords:** hypertension, Mongolian, prevalence, regions, related factors

## Abstract

The objectives of the study were to comprehend the prevalence of hypertension (HTN) and prehypertension (PHT), awareness, treatment, and control of HTN and its distribution in urban, agricultural, pastoral, and semi‐agricultural/semi‐pastoral areas, and to explore the related factors of HTN among Mongolian adults in China. From August 2018 to August 2020, a multi‐stage stratified cluster random sampling method was conducted to investigate the prevalence of HTN among Mongolian adults aged ≥18 years living in China (*n* = 2558). Inclusion criteria for HTN were systolic blood pressure ≥ 140 mm Hg and/or diastolic blood pressure ≥ 90 mm Hg and/or had hypertensive history and/or taking antihypertensive drugs for HTN. The prevalence rates of HTN and PHT were 44.77% and 32.03%, respectively. The prevalence rates of PHT in urban, agricultural, pastoral, and semi‐agricultural/semi‐pastoral areas were 34.93%, 34.73%, 26.03%, and 33.44%, respectively, and the prevalence rates of HTN were 35.97%, 40.15%, 49.68%, and 48.07%, respectively. The awareness, treatment and control rates of HTN were 66.48%, 58.93%, and 16.48%, respectively. In this survey, the overweight, obesity, and central obesity rates were 34.30%, 30.67%, and 58.08%, respectively. Compared with Chinese adults ≥18 years, the prevalence rate of HTN among Mongolian adults in China aged ≥ 18 years was relatively high; the prevalence rate of PHT and HTN awareness, treatment, and control rates were similar. The prevalence of HTN and the rates of obesity and central obesity were higher in pastoral regions than in the other three types of regions, and the rate of overweight was highest in agricultural regions.

## 1 INTRODUCTION

Hypertension (HTN) is a serious challenge all over the world because of the condition's high prevalence in adults and the consequent risk of stroke and cardiovascular disease.^1^ The prevalence of HTN changes with the level of progress of the economy, society, and civilization. According to a systematic analysis, the global age‐standardized prevalence of HTN in 2010 was 31.1%, about three‐quarters of people with HTN live in middle‐ or low‐income countries, and the awareness, treatment, and control rates of HTN were lower in middle‐ and low‐income countries than in high‐income countries.^2^ HTN places a great burden not only on individuals but also on society as a whole. Uncontrolled high blood pressure is reported to lead to premature death and increases heart disease, stroke, kidney failure, blindness, and other complications.^3^ High blood pressure is a risk factor for heart disease,^4^ and there is growing evidence that HTN seems to be associated with common non‐cardiovascular diseases, including dementia, cancer, oral health diseases, and osteoporosis.^5^ In 2017, 2.54 million people died of HTN‐related diseases in China, and 95.7% of these deaths were caused by cardiovascular diseases.^6^

With the continuous reform and opening up seen in China, the country is gradually overcoming poverty, with the population's lives reaching a comfortable level. However, the incidence of non‐infectious chronic diseases is on the rise. According to the results of the Chinese HTN Survey, conducted from 2012 to 2015, the crude prevalence rates of HTN and prehypertension (PHT) among Chinese adults aged ≥ 18 years were 27.9% and 39.1%, respectively.^7^ The prevalence of HTN had increased from 25.2% in 2012 to 27.9% in 2015.^3^ PHT is also known as an independent risk factor for HTN and cardiovascular disease.^8^ However, in 2015, the awareness, treatment, and control rates of HTN among Chinese adults were 51.5%, 46.1%, and 16.9%,^7^ respectively; these numbers were higher than those in previous years, but there was still room for improvement.

HTN is affected by many factors. Some surveys have revealed large differences in the relationship between genetic factors and HTN for people of different races.^9^, ^10^, ^11^ Some studies have shown that Mongolian residents of China have a relatively high prevalence of HTN. From 1991 to 2002, Mongolian were among the ethnic groups with the highest prevalence of HTN in China, and in recent years (2003–2015), Mongolia ranked among the top three countries nationwide in terms of HTN prevalence.^12^ A study conducted by Han on urban and rural areas in Inner Mongolia of China found the prevalence of HTN in Inner Mongolia to be 50.91%.^13^ Chifeng's 2018 study on the Mongolian population and 2019 study on the Tongliao pastoral districts reported the prevalence of HTN as 39.2%^14^ and 39.6%,^15^ respectively.

Historically, Mongolian people in China depended on animal husbandry, with meat and milk as the main foods. With the development of farming in North China, Mongolians’ diet culture, production, and lifestyle changed; the proportion of their diet made up of grains and vegetables gradually increased, and the proportion made up of meat and milk gradually decreased, slowly dividing the Inner Mongolia region into agricultural, pastoral, and part‐farming/part‐pastoral regions.^16^

Most studies on HTN among Mongolian people in China do not distinguish between urban, agricultural, pastoral, and part‐farming/part‐pastoral regions. There are large differences in production, life‐style, and eating habits across these different types of regions, and the impact of these factors on the prevalence of HTN among Mongolia people in China also varies.

This study investigated the prevalence of HTN and PHT, as well as the awareness, treatment, and control rates of HTN among Mongolian adults in China, with particular attention to different by type of region (urban, agricultural, pastoral, or part‐farming/part‐pastoral). We discuss the risk factors for HTN among Mongolian people living in China, which provides valuable information for the prevention and treatment of HTN among this population.

## 2 METHODS

### 2.1 Survey participants

**FIGURE 1:**
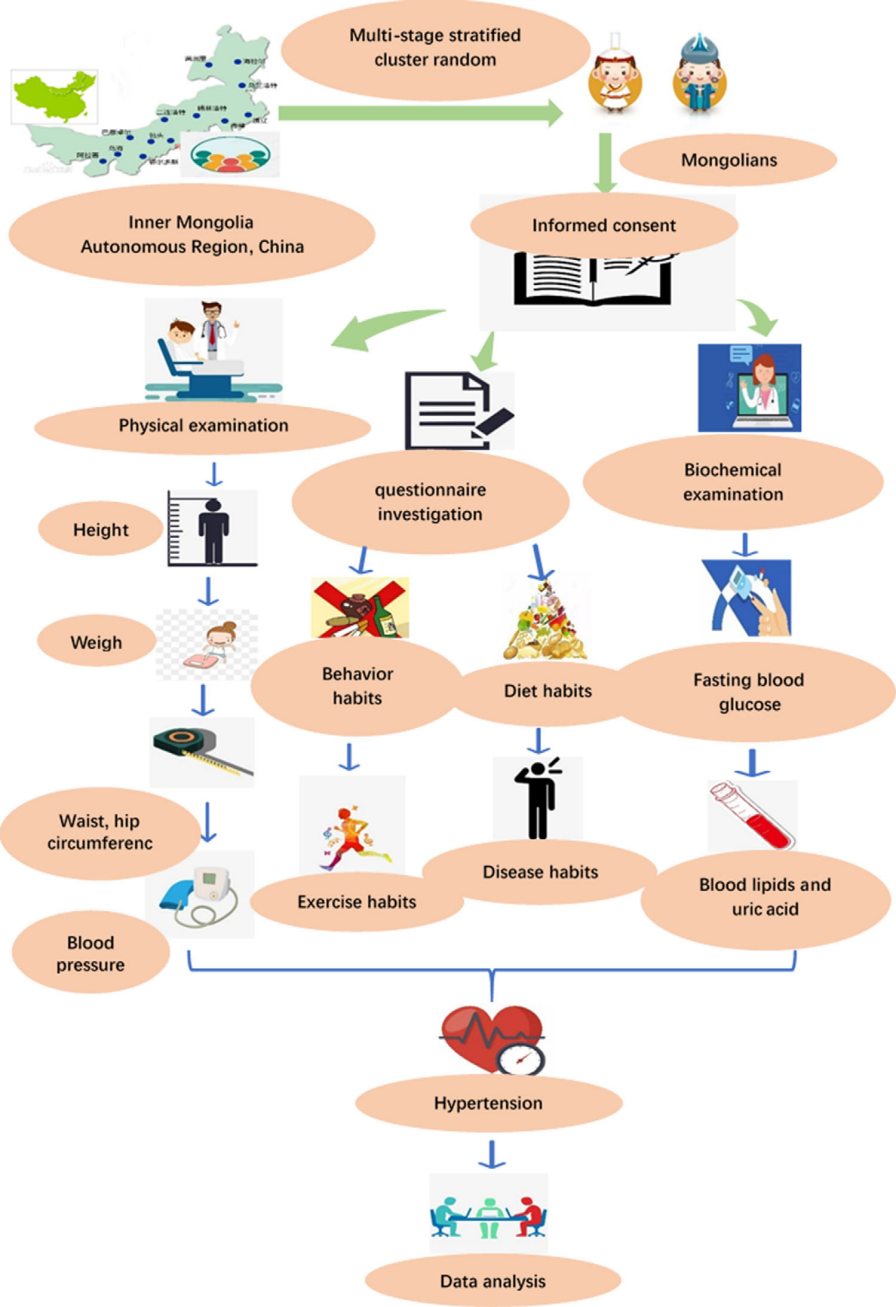
The flowchart of the study

The flowchart of the study is shown in Figure 1. A survey with multi‐stage stratified cluster random sampling was conducted to investigate the prevalence of HTN and PHT among Mongolian adults aged 18 years or older living in the Chinese regions of Tongliao City, Xilinhot City, and Hohhot City on August 7–20, 2018, August 11–26, 2019, and August 12–16, 2020, respectively. Mongolian adult refers to lineal relative by blood up to three generations of the Mongolian nationality. Ultimately, 2558 participants were recruited. Written informed consent was obtained from each participant. The Ethics Committee of Inner Mongolia Medical University (Inner Mongolia, China) approved the study (Approval No.: YKD2016066).

### 2.2 Training and data collection

Before conducting the survey, all investigators participated in a joint training program to standardize the survey methods, unify the measurement standards, and ensure that investigators were familiar with the questionnaire content and with the questionnaire completion instructions. During the survey, the investigators explained the purpose and methods of the survey to the participants and sought to obtain their cooperation to collect accurate data. Blood biochemical tests, a physical examination (measurement of height, weight, waist circumference, hip circumference, and blood pressure), and a questionnaire survey (collect the general information of the participates and 64 food intake history in the past 12 months, including the history of HTN, drug treatment, food intake frequency, and intake per meal, etc.) was conducted for each participant.

### 2.3 Blood pressure measurement

After 5 min of rest, the participants’ blood pressure was measured twice with an interval of 10 min, using an Omron electronic sphygmomanometer (Omron L10; Dalian, China). The mean of the two measurements was used for the analysis. Omron electronic sphygmomanometer was provided by community health services and calibrated regularly.

### 2.4 Outcome definitions

Inclusion criteria for HTN: (1) systolic blood pressure (SBP) ≥ 140 mm Hg and/or diastolic blood pressure (DBP) ≥ 90 mm Hg^3^; (2) hypertensive history; or (3) taking antihypertensive drugs for HTN. PHT referred to SBP of 120–139 mm Hg and/or DBP of 80–89 mm Hg. Dyslipidemia was defined as having any of the following: (1) total cholesterol ≥ 6.2 mmol/L; (2) triglyceride ≥ 2.3 mmol/L; (3) low‐density lipoprotein cholesterol ≥ 4.1 mmol/L; and (4) high‐density lipoprotein cholesterol ≤ 1.0 mmol/L.^17^

Awareness referred to the proportion of all participants categorized as having HTN who reported a history of HTN diagnosed by a doctor. Treatment referred to the proportion of people with high blood pressure who were taking antihypertensive drugs. Control referred to the proportion of people with HTN who had blood pressure < 140/90 mm Hg. Body mass index (BMI) was divided into four classes: ≤ 18.4 kg/m^2^ (underweight), 18.5–23.9 kg/m^2^ (normal weight), 24.0–27.9 kg/m^2^ (overweight), and ≥ 28 kg/m^2^ (obese); central obesity referred to waist circumference ≥ 90 cm for men and ≥ 85 cm for women.^18^

In line with the Chinese guidelines for adult physical activity, physical activity level was divided into three grades: high (≥ 6.0 metabolic equivalents [METs]), medium (3.0–5.9 METs), and low (1.1–2.9 METs).^19^

On the basis of the mode of production, the participants’ regions of residence were categorized as urban, agricultural, pastoral, or semi‐agricultural/semi‐pastoral areas.^20^, ^21^ Urban Mongolians refer to those who live and work in cities. In the agricultural areas, Mongolians are mainly agricultural. The Mongolians in pastoral areas maintain the production and life style based on the traditional animal husbandry economy. The Mongolians in the semi‐agricultural/semi‐pastoral areas are engaged in animal husbandry and agriculture.

### 2.5 Statistical analysis

EpiData 3.1 database software was used to record data from the questionnaires. This error comparison software was used for logical error correction during the input process. At the same time, double‐person input was used to identify and correct errors to ensure the accuracy of the data entry.

Continuous variables are described as means ± standard deviations, and Student's *t*‐test or ANOVA was used for between‐group comparisons. Categorical variables are described as percentages, and the chi‐square test was used for comparisons between groups. The Mantel–Haenszel chi‐square test was used to test trends of linear change. Logistic regression was used for the multivariate analysis. SAS software, Version 9.4 (SAS Institute Inc., Cary, NC, USA) was used to conduct descriptive statistical tests to examine differences between groups. Differences were considered statistically significant when the *p* value was < .05.

## 3 RESULTS

### 3.1 Participants characteristics

In total, 2558 people were included in the survey. Participants with missing data on blood pressure and those of non‐Mongolian ethnicity were excluded. The final analytic sample included data on 2426 participants (19.83% urban, 16.74% agricultural, 25.97% pastoral, and 37.47% part‐farming/part‐pastoral; 38.54% men, and 61.46% women), with an average age of 50.68 ± 14.00 years. The overweight, obesity, and central obesity rates were 34.30%, 30.67%, and 58.08%, respectively. Table 1 shows the basic characteristics of the participants in this survey. Except for calorie group and fat group, significant differences were found in all characteristics across urban, agricultural, pastoral, and part‐farming/part‐pastoral regions. Except for average age, age group, BMI category, central obesity, total cholesterol, low‐density lipoprotein cholesterol, family history of HTN and diabetes, there were significant sex differences in all examined characteristics.

**TABLE 1:** Demographic and clinical characteristics of participants, by sex and regions

Parameters	Total	Regions				*p* value for regions	Sex		*p* value for sex
		Urban	Agricultural	Pastoral	Part‐farming/part‐pastoral		Men	Women	
*N* (%)	2426	481 (19.83)	406 (16.74)	630 (25.97)	909 (37.47)		935 (38.54)	1491 (61.46)	
Age (years) (mean±SD)	50.68±14.00	45.66±16.13	56.36±12.69	51.35±15.11	50.30±11.33	.000	51.17±13.98	50.38±14.01	.176
Age group (*n*, %) (years)									
18–24	106	23 (21.70)	8 (7.55)	61 (57.55)	14 (13.21)	.000	41 (38.68)	65 (61.32)	.655
25–34	240	134 (55.83)	19 (7.92)	25 (10.42)	62 (25.83)		83 (34.58)	157 (65.42)	
35–44	398	100 (25.13)	42 (10.55)	76 (19.10)	180 (45.23)		154 (38.69)	244 (61.31)	
45–54	633	44 (6.95)	85 (13.43)	173 (27.33)	331 (52.29)		242 (38.23)	391 (61.77)	
55–64	638	100 (15.67)	132 (20.69)	181 (28.37)	225 (35.27)		242 (37.93)	396 (62.07)	
≥64	404	73 (18.07)	120 (29.70)	114 (28.22)	97 (24.01)		168 (41.58)	236 (58.42)	
BMI (kg/m^2^) (mean ± SD)									
	26.19±7.82	25.18±4.92	24.69±3.80	27.10±4.99	26.78±10.19	.000	26.59±10.11	25.94±4.79	.034
BMI group (kg/m^2)^ (*n*, %)									
≤18.4	63	18 (28.57)	16 (25.40)	9 (14.29)	20 (31.75)	.000	18 (28.57)	45 (71.43)	.076
18.5–23.9	780	195 (25.00)	162 (20.77)	159 (20.38)	264 (33.85)		280 (35.90)	500 (64.10)	
24.0–27.9	832	158 (18.99)	151 (18.15)	216 (25.96)	307 (36.90)		333 (40.02)	499 (59.98)	
≥28.0	744	109 (14.56)	77 (10.35)	240 (32.26)	318 (42.74)		301 (40.46)	443 (59.54)	
Waist circumference (cm) (mean ± SD)									
	88.93±12.36	85.34±13.08	88.98±9.90	91.92±13.41	88.74±11.70	.000	91.96±11.42	87.02±12.55	.000
Central obesity group (*n*, %)									
Normal	1008	264 (26.19)	161 (15.97)	200 (19.84)	383 (38.00)	.000	374 (37.10)	634 (62.90)	.213
Central obesity	1409	213 (15.12)	245 (17.39)	425 (30.16)	526 (37.33)		558 (39.60)	851 (60.40)	
SBP (mm Hg) (mean ± SD)									
	129.93±21.02	124.24±20.30	127.72±16.77	130.11±19.05	133.81±23.47	.000	133.98±19.19	127.39±21.72	.000
DBP (mm Hg) (mean ± SD)									
	83.98±15.51	82.11±23.21	83.65±10.15	84.62±12.80	84.69±13.97	.017	87.37±12.53	81.86±16.77	.000
Laboratory examination (mean ± SD)									
TC (mmol/L)	4.87±1.09	4.97±0.92	4.61±0.89	5.32±1.11	4.65±1.11	.000	4.84±1.10	4.89±1.08	.311
LDL‐C (mmol/L)	2.84±0.78	2.70±0.68	2.51±0.63	2.87±0.73	3.02±0.85	.000	2.84±0.79	2.84±0.77	.984
HDL‐C (mmol/L)	1.39±0.37	1.48±0.32	1.56±0.31	1.57±0.32	1.14±0.30	.000	1.34±0.36	1.42±0.37	.000
TG (mmol/L)	1.73±1.35	1.70±1.17	1.87±1.59	1.61±1.17	1.77±1.41	.018	1.95±1.69	1.59±1.05	.000
Uric acid (μmol/L)	317.38±95.68	328.11±89.37	365.82±94.36	336.29±92.07	278.55±85.52	.000	366.75±89.86	285.19±85.07	.000
Fast blood glucose (mmol/L) (mean ± SD)									
	5.68±1.58	5.42±1.57	6.11±1.80	5.64±1.39	5.60±1.57	.000	5.77±1.73	5.62±1.48	.032
Calorie (kcal/d) (mean ± SD)									
	1839.04±3303.50	1564.55±1145.24	1689.25±1115.74	1777.26±703.05	1731.58±1701.54	.079	1917.41±2476.13	1617.74±936.48	.000
Nutrients (g/d) (mean ± SD)									
Protein	94.83±102.16	103.88±104.76	81.88±101.44	112.62±132.57	83.10±68.76	.000	113.91±132.42	83.14±75.70	.000
Fat	51.65±75.16	56.20±147.55	49.66±51.53	52.08±42.91	49.81±33.33	.457	58.05±109.54	47.74±41.41	.001
Carbohydrate	248.96±232.59	262.22±444.14	220.50±155.76	218.49±148.58	275.87±110.81	.000	273.10±332.81	234.16±137.06	.000
Total fiber	13.06±9.03	14.39±11.75	12.43±8.21	12.85±9.33	12.78±7.26	.003	13.97±10.78	12.50±7.71	.000
Level of education (*n*, %)									
Primary school or below	1122	107 (9.54)	168 (14.97)	270 (24.06)	577 (51.43)	.000	376 (33.51)	746 (66.49)	.000
Secondary school	533	47 (8.82)	129 (24.20)	171 (32.08)	186 (34.90)		244 (45.78)	289 (54.22)	
High school or above	765	326 (42.61)	108 (14.12)	188 (24.58)	143 (18.69)		315 (41.18)	450 (58.82)	
Smoking status (*n*, %)									
No	1901	421 (22.15)	268 (14.10)	515 (27.09)	697 (36.66)	.000	559 (29.41)	1342 (70.59)	.000
Yes	524	60 (11.45)	138 (26.34)	115 (21.95)	211 (40.27)		376 (71.76)	148 (28.24)	
Consumption of alcohol (*n*, %)									
No	1615	249 (15.42)	275 (17.03)	479 (29.66)	612 (37.89)	.000	452 (27.99)	1163 (72.01)	.000
Yes	811	232 (28.61)	131 (16.15)	151 (18.62)	297 (36.62)		483 (59.56)	328 (40.44)	
Activity status (*n*, %)									
Low intensity	885	82 (9.27)	107 (12.09)	168 (18.98)	528 (59.66)	.000	302 (34.12)	583 (65.88)	.000
Medium strength	985	301 (30.56)	232 (23.55)	278 (28.22)	174 (17.66)		333 (33.81)	652 (66.19)	
High strength	556	98 (17.63)	67 (12.05)	184 (33.09)	207 (37.23)		300 (53.96)	256 (46.04)	
Working status (*n*, %)									
No	457	55 (12.04)	127 (27.79)	122 (26.70)	153 (33.48)	.000	127 (27.79)	330 (72.21)	.000
Yes	1960	421 (21.48)	279 (14.23)	506 (25.82)	754 (38.47)		804 (41.02)	1156 (58.98)	
Marriage status (*n*, %)									
Unmarried	218	91 (41.74)	17 (7.80)	79 (36.24)	31 (14.22)	.000	81 (37.16)	137 (62.84)	.000
Married or cohabiting	1949	330 (16.93)	369 (18.93)	461 (23.65)	789 (40.48)		799 (41.00)	1150 (59.00)	
Widowed, divorced or separated	248	57 (22.98)	20 (8.06)	89 (35.89)	82 (33.06)		51 (20.56)	197 (79.44)	
Family history of hypertension (*n*, %)									
No	1213	237 (19.54)	230 (18.96)	390 (32.15)	356 (29.35)	.000	486 (40.07)	727 (59.93)	.123
Yes	1213	244 (20.12)	176 (14.51)	240 (19.79)	553 (45.59)		449 (37.02)	764 (62.98)	
Family history of diabetes (*n*, %)									
No	2127	419 (19.70)	348 (16.36)	588 (27.64)	772 (36.30)	.000	814 (38.27)	1313 (61.73)	.456
Yes	299	62 (20.74)	58 (19.40)	42 (14.05)	137 (45.82)		121 (40.47)	178 (59.53)	

*Abbreviations*: BMI, body mass index; DBP, diastolic blood pressure; HDL‐C, high‐density lipoprotein cholesterol; LDL‐C, low‐density lipoprotein cholesterol; SBP, systolic blood pressure; TC, total cholesterol; TG, triglycerides.

### 3.2 Prevalence of HTN and PHT

Survey data from a 2019 national sample were used to standardize the prevalence of HTN and PHT in the research population, with a sampling ratio of 0.780 to 1000 population.^22^ As Table 2 shows, the prevalence rates of HTN and PHT were 44.77% and 32.03%, respectively (standardized rates: 35.50% and 34.99%, respectively), and men had higher prevalence than did women (50.91% vs. 40.91% for HTN, 35.40% vs. 29.91% for PHT, *p *< .05). The prevalence of HTN differed significantly by all examined variables (*p *< .05) except smoking, family history of diabetes, calorie group, fat group, carbohydrate group, and total fiber group. The prevalence of HTN increased with age and BMI (*p* < .05) and was higher for those with central obesity than for those with normal waist circumference (*p* < .05). The prevalence of PHT differed significantly by all examined variables (*p *< .05) except family history of diabetes, calorie group, protein group, fat group, carbohydrate group, and total fiber group. The prevalence of PHT was the highest in the 25–34 years age group (45.83%), in the 18.5–23.9 kg/m^2^ BMI group (35.64%), in the normal waist circumference group (36.21%), and in the urban group (34.93%).

**TABLE 2:** Prevalence of prehypertension and hypertension in Mongolian adults in China

Parameters	Total	Normal (*n*, %)	Prehypertension (*n*, %)	*p* value for prehypertension	Hypertension (*n*, %)	*p* value for hypertension
Total	2426	563 (23.21)	777 (32.03)		1086 (44.77)	
Sex (*n*, %)						
Men	935	128 (13.69)	331 (35.40)	.005	476 (50.91)	<.000
Women	1491	435 (29.18)	446 (29.91)		610 (40.91)	
Age (years) (*n*, %)						
18–24	106	54 (50.94)	45 (42.45)	<.000	7 (6.60)	<.000
25–34	240	108 (45.00)	110 (45.83)		22 (9.17)	
35–44	398	154 (38.69)	146 (36.68)		98 (24.62)	
45–54	633	121 (19.12)	215 (33.97)		297 (46.92)	
55–64	638	76 (11.91)	167 (26.81)		395 (61.91)	
≥64	404	50 (12.38)	89 (22.03)		265 (65.59)	
*p* ^a^ value			<.0001		<.0001	
BMI (kg/m^2)^ (*n*, %)						
≤18.4	63	37 (58.73)	15 (23.81)	.002	11 (17.46)	<.000
18.5–23.9	780	266 (34.10)	278 (35.64)		236 (30.26)	
24.0–27.9	832	179 (21.51)	280 (33.65)		373 (44.83)	
≥28.0	744	81 (10.89)	203 (27.28)		460 (61.83)	
*p* ^a^ value			.0069		<.0001	
Central obesity group (*n*, %)						
Normal	1008	357 (35.42)	365 (36.21)	.000	286 (28.37)	<.000
Central obesity	1409	204 (14.48)	411 (29.17)		794 (56.35)	
Region (*n*, %)						
Urban	481	140 (29.11)	168 (34.93)	.002	173 (35.97)	<.000
Agricultural	406	102 (25.12)	141 (34.73)		163 (40.15)	
Pastoral	630	153 (24.29)	164 (26.03)		313 (49.68)	
Part‐farming/part‐pastoral	909	168 (18.48)	304 (33.44)		437 (48.07)	
T2DM (*n*, %)						
No	2155	519 (24.08)	711 (32.99)	.004	925 (42.92)	<.000
Yes	271	44 (16.24)	66 (24.35)		161 (59.41)	
Dyslipidemia (*n*, %)						
No	1386	405 (29.22)	488 (35.21)	.000	493 (35.57)	<.000
Yes	1040	158 (15.19)	289 (27.79)		593 (57.02)	
Family history of hypertension (*n*, %)						
No	1213	287 (23.66)	415 (34.21)	.021	511 (42.13)	.009
Yes	1213	276 (22.75)	362 (29.84)		575 (47.40)	
Family history of diabetes (*n*, %)						
No	2127	489 (22.99)	683 (32.11)	.815	955 (44.90)	.724
Yes	299	74 (24.75)	94 (31.44)		131 (43.81)	
Calorie (kcal/d) (mean ± SD)	1731.58±1701.53	1734.88±1109.30	1723.85±896.74	.841^b^	1734.79±2282.33	.999^b^
Nutrients (g/d) (mean ± SD)						
Protein	94.83±102.16	99.93±96.44	99.60±109.62	.954^b^	88.84±99.98	.024^b^
Fat	51.65±75.16	51.60±48.32	49.96±35.20	.475^b^	52.79±102.05	.785^b^
Carbohydrate	248.96±232.59	250.93±159.32	251.96±136.35	.899^b^	245.89±305.99	.703^b^
Total fiber	13.06±9.03	13.26±7.97	13.44±8.77	.709^b^	12.70±9.72	.218^b^
Level of education (*n*, %)						
Primary school or below	1122	184 (16.40)	320 (28.52)	.001	618 (55.08)	<.000
Secondary school	533	141 (26.45)	176 (33.02)		216 (40.53)	
High school or above	765	238 (31.11)	279 (36.47)		248 (32.42)	
Smoking status (*n*, %)						
No	1901	472 (24.83)	582 (30.62)	.000	847 (44.56)	.667
Yes	524	91 (17.37)	194 (37.02)		239 (45.61)	
Consumption of alcohol (*n*, %)						
Yes	811	180 (22.19)	296 (36.50)	.000	335 (41.31)	.015
No	1615	383 (23.72)	481 (29.78)		751 (46.50)	
Activity status (*n*, %)						
Low intensity	885	197 (22.26)	275 (31.07)	.000	413 (46.67)	<.000
Medium strength	985	214 (21.73)	285 (28.93)		486 (49.34)	
High strength	556	152 (27.34)	217 (39.03)		187 (33.63)	
Working status (*n*, %)						
No	457	77 (16.85)	119 (26.04)	<.000	261 (57.11)	<.000
Yes	1960	485 (24.74)	653 (33.32)		822 (41.94)	
Marriage status (*n*, %)						
Unmarried	218	105 (48.17)	90 (41.28)	<.000	23 (10.55)	<.000
Married or cohabiting	1949	424 (21.75)	626 (32.12)		899 (46.13)	
Widowed, divorced or separated	248	33 (13.31)	54 (21.77)		161 (64.92)	

*Abbreviations*: BMI, body mass index; DBP, diastolic blood pressure; HDL‐C, high‐density lipoprotein cholesterol; LDL‐C, low‐density lipoprotein cholesterol; SBP, systolic blood pressure; T2DM,  type 2 diabetes; T2DM, Type 2 Diabetes Mellitus; TC, total cholesterol; TG, triglycerides.

^a^ Mantel Haenszel chi‐square test.

^b^ Compared with the normal group.

### 3.3 Awareness, treatment, and control of HTN

The results on the awareness, treatment, and control of HTN are shown in Table 3. Of the 1086 participants with HTN, 722 knew about their condition, 640 were taking antihypertensive drugs, and 179 kept their blood pressure at a normal level. The awareness, treatment, and control rates of HTN were 66.48%, 58.93%, and 16.48%, respectively. There were statistically significant differences in awareness rate by all examined variables except smoking and family history of diabetes. There were significant differences in treatment rate by all examined variables except sex, smoking, and family history of diabetes. Control rate showed statistically significant differences by all examined variables except BMI groups, smoking, family history of diabetes and HTN. The rates of HTN awareness, treatment, and control were higher for women than for men (69.18% vs. 63.03%, 62.46% vs. 54.41%, and 20.33% vs. 11.55%, respectively) and these rates were highest in the ≥ 65 years age group (80.00%, 74.34%, and 25.66%, respectively). These rates increased with BMI (P < .05), and the awareness, treatment, and control rates were also higher in the central obesity group than in the normal waist circumference group (70.03% vs. 57.34%, 62.97% vs. 48.25%, and 16.62% vs. 16.08%, respectively; *P* < .05).

**TABLE 3:** Awareness, treatment, and control of hypertension in Mongolian adults in China

Parameters	Hypertension	Awareness, *n* (%)	*p* value for awareness	treatment, *n* (%)	*p* value for treatment	control, *n* (%)	*p* value for control
Total	1086	722 (66.48)		640 (58.93)		179 (16.48)	
Sex							
Men	476	300 (63.03)	.047	259 (54.41)	.243	55 (11.55)	.026
women	610	422 (69.18)		381 (62.46)		124 (20.33)	
Age (years)							
18–24	7	3 (42.86)	<.000	1 (14.29)	<.000	1 (14.29)	<.000
25–34	22	4 (18.18)		2 (9.09)		1 (4.55)	
35–44	98	40 (40.82)		30 (30.61)		7 (7.14)	
45–54	297	175 (58.92)		157 (52.86)		29 (9.76)	
55–64	395	287 (72.66)		252 (63.80)		72 (18.23)	
≥65	265	212 (80.00)		197 (74.34)		68 (25.66)	
*p* ^a^ value		<.0001		<.0001		<.0001	
BMI (kg/m^2)^							
≤18.4	11	6 (54.55)	<.000	6 (54.55)	<.000	3 (27.27)	.149
18.5–23.9	236	153 (64.83)		124 (52.54)		51 (21.61)	
24.0–27.9	373	231 (61.93)		208 (55.76)		56 (15.01)	
≥28.0	460	330 (71.74)		300 (65.22)		68 (14.78)	
*p* ^a^ value		<.0001		<.0001		.037	
Central obesity group							
Normal	286	164 (57.34)	<.000	138 (48.25)	<.000	46 (16.08)	<.000
Central obesity	794	556 (70.03)		500 (62.97)		132 (16.62)	
Region							
Urban	173	123 (71.10)	<.000	108 (62.43)	<.000	36 (20.81)	.001
Agricultural	163	113 (69.33)		96 (58.90)		34 (20.86)	
Pastoral	313	243 (77.64)		222 (70.93)		64 (20.45)	
Part‐farming/part‐pastoral	437	243 (55.61)		214 (48.97)		45 (10.30)	
T2DM							
No	925	592 (64.00)	<.000	524 (56.65)	<.000	146 (15.78)	.001
Yes	161	130 (80.75)		116 (72.05)		33 (20.50)	
Dyslipidemia							
No	493	301 (61.05)	<.000	263 (53.35)	<.000	74 (15.01)	<.000
Yes	593	421 (70.99)		377 (63.58)		105 (17.71)	
Family history of hypertension							
No	511	328 (64.19)	.003	290 (56.75)	.006	91 (17.81)	.816
Yes	575	394 (68.52)		350 (60.87)		88 (15.30)	
Family history of diabetes							
No	955	637 (66.70)	.590	564 (59.06)	.687	152 (15.92)	.243
Yes	131	85 (64.89)		76 (58.02)		27 (20.61)	
Level of education							
Primary school or below	618	428 (69.26)	<.000	391 (63.27)	<.000	120 (19.42)	<.000
Secondary school	216	143 (66.20)		120 (55.56)		25 (11.57)	
High school or above	248	148 (59.68)		127 (51.21)		34 (13.71)	
Smoking status							
No	847	571 (67.41)	.589	507 (59.86)	.554	145 (17.12)	.377
Yes	239	151 (63.18)		133 (55.65)		34 (14.23)	
Consumption of alcohol							
No	751	534 (71.11)	<.000	485 (64.58)	<.000	140 (18.64)	<.000
Yes	335	188 (56.12)		155 (46.27)		39 (11.64)	
Activity status							
Low intensity	413	254 (61.50)	<.000	225 (54.48)	<.000	57 (13.80)	<.000
Medium strength	486	353 (72.63)		318 (65.43)		100 (20.58)	
High strength	187	115 (61.50)		97 (51.87)		22 (11.76)	
Working status							
No	261	190 (72.80)	<.000	166 (63.60)	<.000	54 (20.69)	<.000
Yes	822	529 (64.36)		471 (57.30)		125 (15.21)	
Marriage status							
Unmarried	23	11 (47.83)	<.000	7 (30.43)	<.000	6 (26.09)	<.000
Married or cohabiting	899	588 (65.41)		520 (57.84)		136 (15.13)	
Widowed, divorced or separated	161	121 (75.16)		111 (68.94)		37 (22.98)	

*Abbreviations*: BMI, body mass index; DBP, diastolic blood pressure; HDL‐C, high‐density lipoprotein cholesterol; LDL‐C, low‐density lipoprotein cholesterol; SBP, systolic blood pressure; T2DM,  type 2 diabetes; T2DM, Type 2 Diabetes Mellitus; TC, total cholesterol; TG, triglycerides.

^a^ Mantel Haenszel chi‐square test.

### 3.4 Prevalence of HTN and PHT, overweight, obesity, and central obesity in urban, agricultural, pastoral, and part‐farming/part‐pastoral regions

**FIGURE 2:**
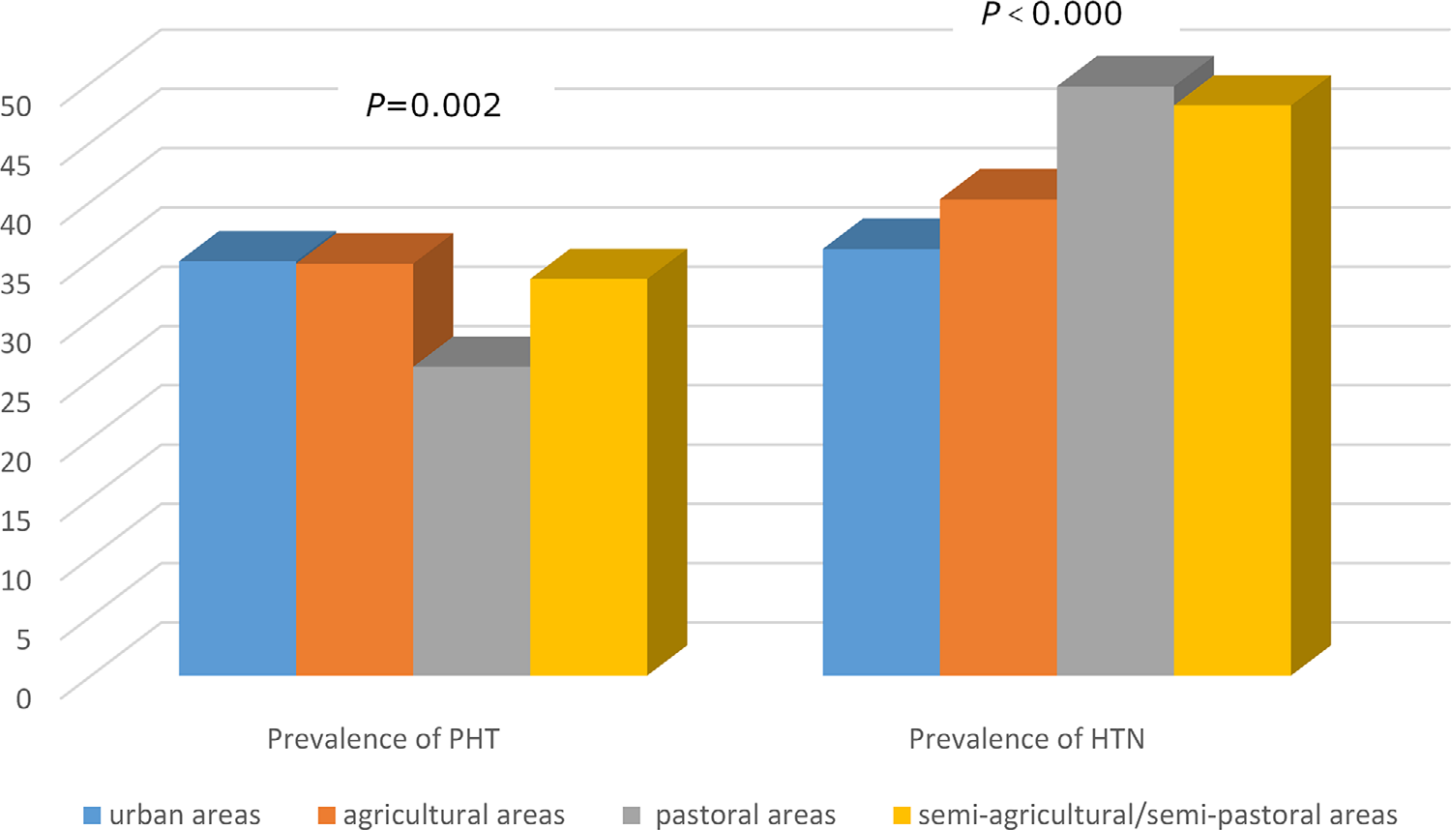
Prevalence of PHT and HTN in urban, agricultural, pastoral, and semi‐agricultural/semi‐pastoral areas

**FIGURE 3:**
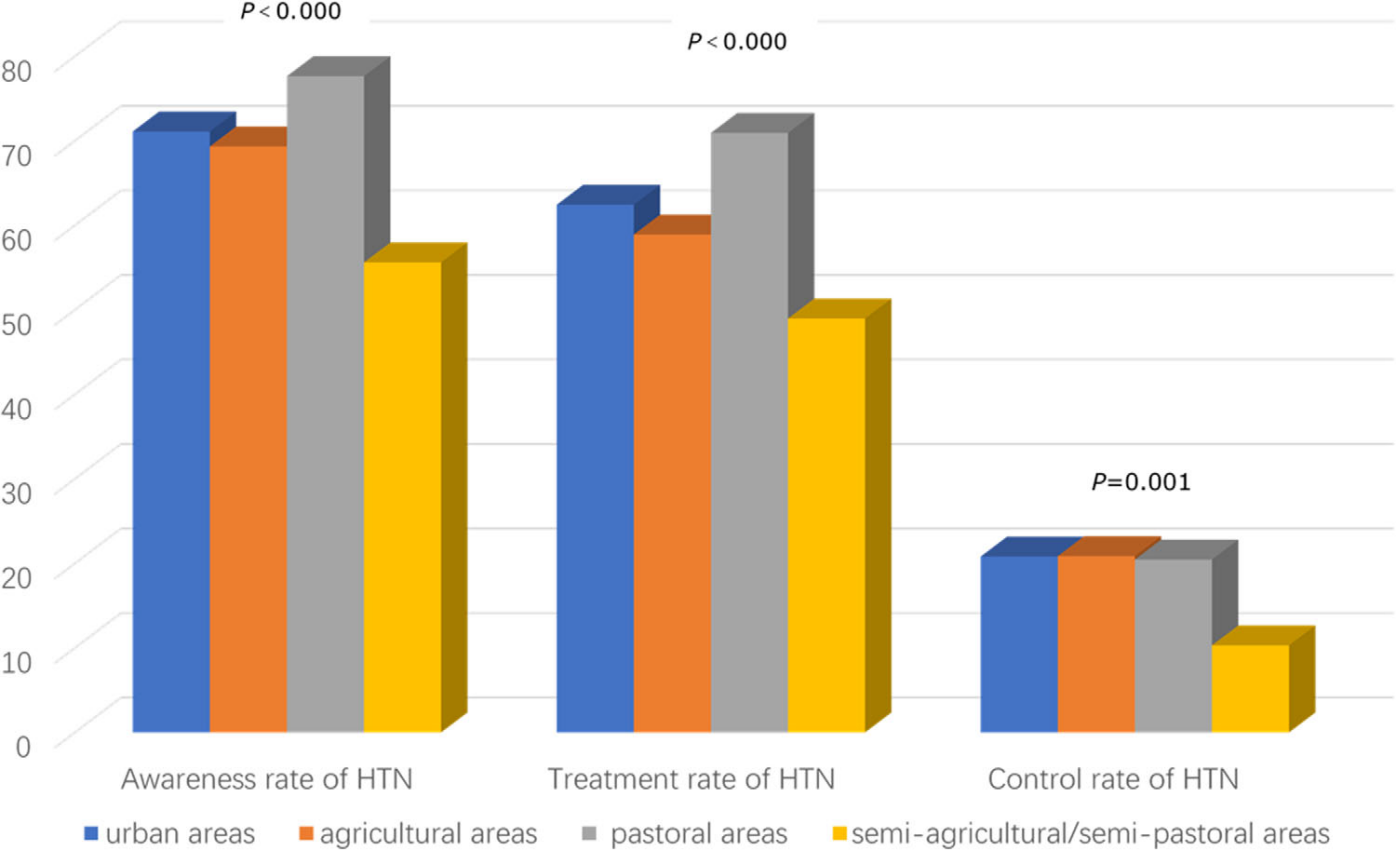
Awareness, treatment, and control rates of HTN in urban, agricultural, pastoral, and semiagricultural/semi‐pastoral

**FIGURE 4:**
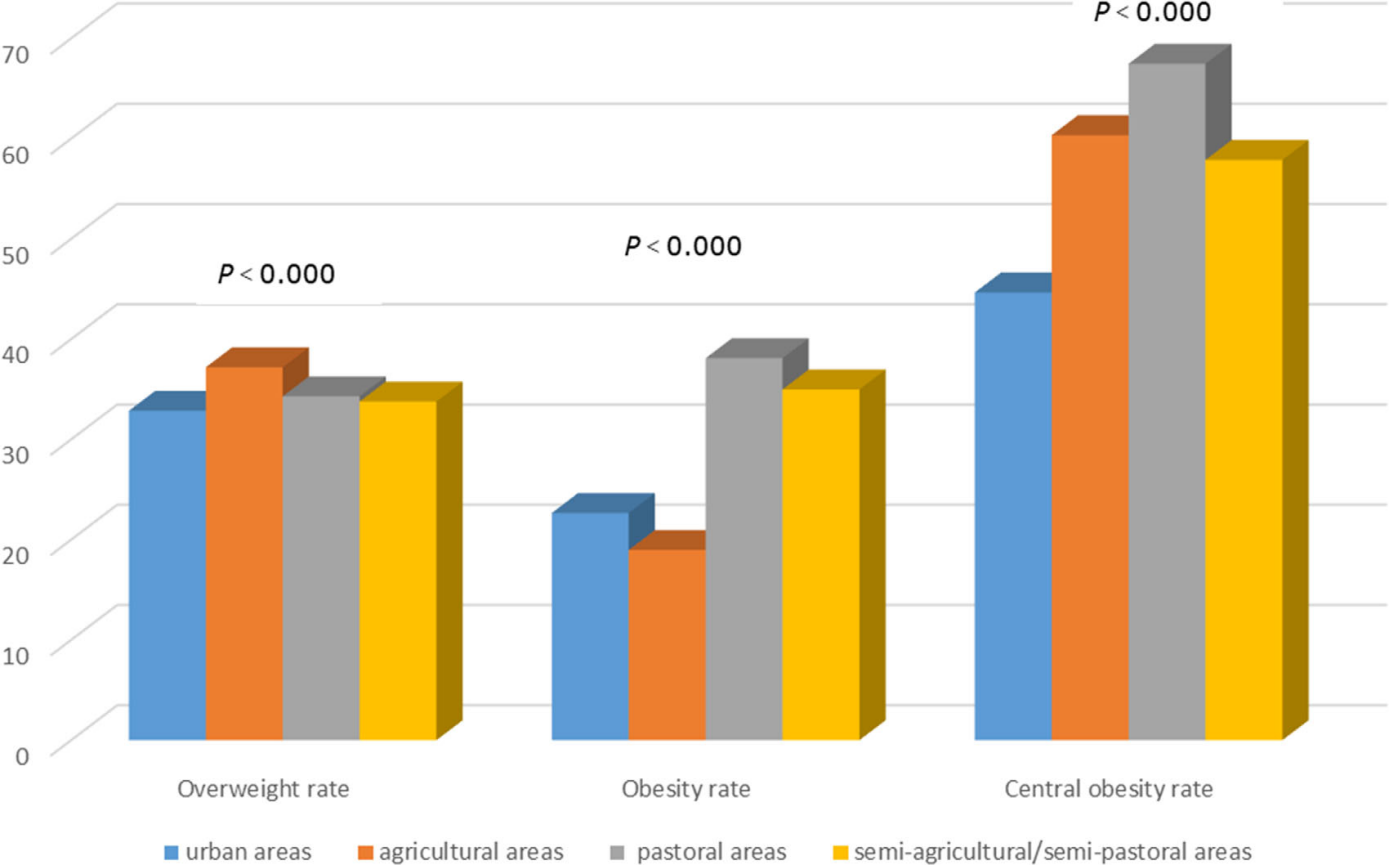
Overweight, obesity, and central obesity rates in urban, agricultural, pastoral, and semiagricultural/semi‐pastoral areas

In urban, agricultural, pastoral, and part‐farming/part‐pastoral regions, the prevalence of PHT was 34.93%, 34.73%, 26.03%, and 33.44%, respectively, and the prevalence of HTN was 35.97%, 40.15%, 49.68%, and 48.07%, respectively. People living in pastoral regions had a higher HTN prevalence compared with those in other three regions (*p *< .05). The overweight rate was 32.85% in urban, 37.19% in agricultural, 34.29% in pastoral, and 33.77% in part‐farming/part‐pastoral regions. Obesity was found among 22.66% of urban‐region participants, 18.97% of agricultural‐region participants, 38.10% of pastoral‐region participants, and 34.98% of part‐farming/part‐pastoral‐region participants. The central obesity rate was 44.28%, 60.34%, 67.46%, and 57.87% in urban, agricultural, pastoral, and part‐farming/part‐pastoral regions, respectively. In urban, agricultural, pastoral, and part‐farming/part‐pastoral regions, the HTN awareness rate was 71.10%, 69.33%, 77.64%, and 55.61%, respectively; the treatment rate was 62.43%, 58.90%, 70.93%, and 48.97%, respectively; and the control rate was 20.81%, 20.86%, 20.45%, and 10.30%, respectively (Figures 2–4). The awareness and treatment rates were higher in pastoral regions than in the other three types of regions (*p *< .05), and the control rate was higher in agricultural regions than in the other three types of regions (*p *< .05).

### 3.5 Multivariable risk assessment

Age ≥35 years, overweight, obesity, central obesity, male sex, dyslipidemia, and family history of HTN were risk factors for HTN (*p *< .05). Residence in agricultural areas and physical activity were protective factors for HTN (*p *< .05), as shown in Table 4.

**TABLE 4:** Factors associated with the prevalence of hypertension in Mongolian adults in China

Parameters	*β*	*S.E*.	*Wald*	*p* value	*Odds ratio* (OR)	OR 95% *CI*
Age (years)			242.119	.000		
18–24	−0.081	0.491	0.027	.868	0.922	0.352,2.411
25–34					1	
35–44	1.105	0.268	17.035	.000	3.019	1.787,5.103
45–54	2.083	0.257	65.840	.000	8.029	4.854,13.279
55–64	2.731	0.258	112.098	.000	15.350	9.258,25.449
≥64	2.983	0.270	122.016	.000	19.749	11.632,33.528
BMI (kg/m^2^)			36.922	.000		
≤18.4	−0.646	0.387	2.786	.095	0.524	0.246,1.119
18.5–23.9					1	
24.0–27.9	0.290	0.138	4.419	.036	1.337	1.020,1.753
≥28.0	0.868	0.162	28.434	.000	2.382	1.733,3.273
Sex	0.532	0.101	27.820	.000	1.703	1.397,2.075
Central obesity group	0.286	0.134	4.579	.032	1.332	1.024,1.731
Region			19.514	.000		
Urban					1	
Agricultural	−0.594	0.172	11.939	.001	0.552	0.394,0.773
Pastoral	−0.011	0.160	0.005	.944	0.989	0.722,1.353
Part‐farming/par‐pastoral	−0.007	0.158	0.002	.965	0.993	0.729,1.352
Dyslipidemia	0.346	0.101	11.758	.001	1.413	1.198,1.774
Family history of hypertension	0.341	0.099	11.970	.001	1.407	1.159,1.707
Activity status			9.393	.009		
Low intensity	−0.228	0.118	3.739	.053	0.796	0.632,1.003
Medium strength					1	
High strength	−0.413	0.138	8.943	.003	0.662	0.505,0.867

*Abbreviation*: BMI, body mass index.

## 4 DISCUSSION

A total of 2426 Mongolian adults aged 18 years or older living in China participated in this survey. The prevalence rates of HTN and PHT were 44.77% (standardized prevalence: 35.50%; women: 40.91%, men: 50.91%) and 32.03% (standardized prevalence: 34.99%; women: 29.91%, men 35.40%), respectively. The awareness, treatment, and control rates of HTN were 66.48%, 58.93%, and 16.48%, respectively.

The prevalence of HTN among Mongolians in China in this survey was 44.77%, which was higher than the prevalence in Beijing (35.9%), Tianjin (34.5%), and Shanghai (29.1%), the most developed provinces in China.^7^ The HTN prevalence seen among Mongolians in China was also higher than that found in some developed countries, including that found among US adults (29.1%)^23^ and in Canada (19.5%), England (30%),^24^ and Spain (42.6%).^25^ The overall HTN prevalence for Mongolians in China was also higher than the prevalence reported for other ethnic minorities in China, such as Tibetans (36%)^26^ and Uyghurs (15.73%).^27^ In our study, the prevalence of HTN was observed to increase with age and BMI and to be higher for men than for women, which is in line with previous studies.^28^ Existing studies have shown that HTN is associated with BMI, family history, waist–hip ratio, chronic disease history, and eating habits.^29^ In the past 18 years, the prevalence of obesity in China has increased significantly.^30^ High BMI is a risk factor for HTN, and previous studies have demonstrated a quantitative relationship between BMI and HTN.^31^ Overweight affects the incidence of HTN.^32^ Overweight and obesity are associated with a remarkable increase in the hazard of all‐cause death in the world's population^33^ and are also major risk factors for HTN. In the survey in the present study, the rates of overweight, obesity, and central obesity were 34.30%, 30.67%, and 58.08%, respectively. For overweight and obesity, these rates are higher than the respective rates of 31.6% and 12.1% among Chinese adults in 2015^7^ and 14.6% and 3.4% among Indian adults in 2016.^34^ Weight control is essential in the prevention and management of HTN.

The prevalence of PHT in this survey was 32.03%, which is similar to previous findings of 32.30% in Jiangxi Province and 32.10% in Zhejiang Province^35^ and lower than previous finding of 36.00% in northern China,^36^ 34.00% among adults in Taiwan,^37^ 36.10% among Brazilian adults,^38^ 43.9% among adults in England, 36.0% among adults in USA,^24^ and 35.4% among Nepal adults.^39^ Our finding for the prevalence of PHT in pastoral regions (26.03%) was lower than that found among Mongolians in China as a whole. Urban (34.93%), agricultural (34.73%), and semi‐agricultural/semi‐pastoral (33.44%) regions had higher prevalence of PHT compared with the overall region. Some previous studies have shown that PHT is more common among men than among women,^40^ which is consistent with the results of the present study. Numerous studies have shown that PHT is associated with cardiovascular and cerebrovascular events, diabetes, and organ damage and that, compared with those with normal blood pressure, people with PHT are more likely to develop HTN.^41^ To manage the condition of the HTN‐prone population, comprehensive lifestyle interventions, including nutritional guidance, exercise recommendations, psychological support, and smoking cessation programs are needed.^6^

We found that the prevalence of HTN was relatively high in urban, agricultural, pastoral, and part‐farming/part‐pastoral regions. The overweight rate was higher in agricultural regions than in the other three types of regions, and the obesity and central obesity rates were highest in pastoral regions. Thus, the highest rates of overweight and obesity were found in agricultural and pastoral regions, respectively, and the highest prevalence rates of PHT and HTN were observed in urban and pastoral regions, respectively. However, the prevalence rates of PHT found in urban and rural regions were very close. Differences in HTN prevalence are affected by regional variation in topography, eating habits, living habits and genetic factors. Body composition differences have been observed between Chinese Mongolians and people of other nationalities in China, which is related to genetic and environmental factors.^42^ Some studies have found that specific food group consumption patterns are significantly associated with HTN.^43^ In the present study, exercise was a protective factor for HTN among Mongolian people living in China. Proper exercise can prevent HTN because physical exercise can improve the blood supply in the human body and make the circulatory system function well.^44^ A previous study reported that the prevalence of HTN declined as education increased and that, compared with the general population, the hazard of HTN was about twice as high among people with diabetes, about 50% higher among people with dyslipidemia, and about twice as high among people with a family history of HTN.^45^ A different study found that, compared with nonsmokers, smokers had a higher risk of HTN.^15^ Long‐term and excessive drinking can also lead to elevated blood pressure.^46^, ^47^ However, in the present study, we did not find drinking or smoking to have an effect on HTN, which may be related to the diet and living habits of Mongolian people in China.

In China, the rates of HTN awareness, treatment, and control, respectively, were 30.2%, 24.7%, and 6.1% in 2002; 46.5%, 41.1%, and 13.8% in 2012; and 51.5%, 46.1%, and 16.9% in 2015.^7^ A previous study of Mongolian people in China reported the rates of HTN awareness, treatment, and control to be 43.5%, 31.6%, and 8.3%, respectively.^48^ In the present survey, these three rates among Mongolian people living in China were found to be close to the national level. The rates of HTN awareness, treatment, and control are gradually increasing among Mongolian people in China, which is related to the great progress of Healthcare Quality and Access Index since 1990.^49^ The rates of HTN awareness, treatment, and control in urban, agricultural, pastoral, and part‐farming/part‐pastoral regions in this study were similar to the national Chinese level but higher than those previously reported among Tibetans (45%, 30%, and 7%)^26^ and Uyghurs (59.57%, 52.74%, and 21.29%)^27^ in China. These differences are closely related to regional variation in economy and culture. Poor medical conditions, lack of health service personnel, and insufficient service ability and health education in remote areas all affect the control of HTN. Our finding suggested that, among Mongolian people living in China, the rates of HTN treatment, awareness, and control were higher in pastoral than in the other three types of regions, indicating that the attention to pastoral regions has been gradually improved in recent years. However, the rates of HTN awareness, treatment, and control found among Mongolian people in China in this survey were lower than those reported for developed countries such as the United States, where these rates are 84.2%, 77.9%,^50^ and 43.7%,^51^ respectively. In our study, these three rates were highest in the ≥ 65 years age group. A previous study showed that patients aged over 50 years were more likely to be affected by HTN and related complications and that physical discomfort and economic burden are likely to encourage individuals to understand HTN‐related knowledge and health education, enhancing their health awareness and self‐management ability.^52^ This is consistent with the results of our study.

Meat, grain, and milk are the main food in urban and semi‐agricultural/semi‐pastoral areas; the Mongolian diet in the agricultural areas is mainly grain, the Mongolian diet in pastoral areas is based on meat and milk.^21^ In contrast, the intake of dietary fiber in pastoral areas is less. A survey in the United States found that total fiber intake, cereal, and plant fiber intake were associated with reducing the risk of high blood pressure.^53^ A study has shown that a protein diet can lower blood pressure compared to a standard, high‐carbohydrate Dietary Approaches to Stop HTN diet.^54^ The HTN group in this study had lower protein intake than the normal group. A survey has shown that dietary structure^55^ affects the occurrence of chronic diseases in different regions of China. However, the influence of dietary structure in different regions on the prevalence of HTN in Mongolians needs further study.

The Global Heart Initiative has developed five programs to promote cardiovascular health, including the MPOWER package to control tobacco, the ACTIVE package to increase physical activity, the SHAKE package to reduce salt, the REPLACE package to eliminate industrially produced trans fats from the global food supply, and the HEARTS technology package to strengthen the management of central vascular disease in primary health care.^56^ China has developed a blood pressure health management path and assessment system for the whole population, providing a full range of blood pressure health management service guidance, including health information collection, screening and evaluation, dietary guidance, exercise intervention, psychological counseling and drug therapy.^18^

In general, for the Mongolian in Inner Mongolia Autonomous Region in China, the community should strengthen the relevant publicity activities, publicize the harm of HTN, and how to prevent HTN by changing lifestyle and eating habits, improve the compliance of patients with medication, improve prognosis, strengthen the management of HTN patients, reduce the incidence of HTN, and improve the rates of awareness, treatment, and control of HTN.

### 4.1 Limitations

This survey had several limitations. First, the study was completed in three consecutive years; however, all surveys were administered by the same team and in the same calendar month each year. Second, there were more middle‐aged people in this sample than in the overall population; however, because we standardized the prevalence of HTN to a 2019 national sample, the representativeness of our finding is very strong. Third, because this research relied on a cross‐sectional survey, the ability to infer causality is weak, and it was therefore difficult to explore the causal relationships between various factors and HTN.

## 5 CONCLUSIONS

In conclusions, compared with the national level, the results of this cross‐sectional survey showed that the prevalence of HTN was higher among Mongolian adults aged ≥18 years in China; the rates of obesity, overweight and central obesity were relatively high; the prevalence of PHT, the awareness, treatment, and control rates of HTN were similar, but the overall rate was low. The prevalence of HTN and the rates of obesity and central obesity were higher in pastoral regions than in the other three types of regions, and the rates of overweight was highest in agricultural regions. This survey and previous research have demonstrated that HTN has become an important public health problem threatening the Mongolian people in China.

## CONFLICT OF INTEREST

All authors report no potential conflicts of interest in relation to this article.

## AUTHOR CONTRIBUTIONS

Lingyan Zhao contributed to the conception and design of the work. Peiyao Yu and Yuzhen Ning prepared the first draft of the manuscript. Yumin Gao, Yanping Zhao, Lin Tie, Lijitu Wu, Lili Zhang, and Ru Zhang contributed to the collection of data for the work. Meng Cui, Hui Pang, Qian Wu, Zhidi Wang, and Le Chen contributed to data analysis and interpretation for the work. All authors critically revised the manuscript and gave the final approval.
